# Experience with a new prosthetic anal sphincter in three coloproctological centres

**DOI:** 10.1186/1471-2482-13-45

**Published:** 2013-10-08

**Authors:** Matthias Goos, Ulrich Baumgartner, Mathias Löhnert, Oliver Thomusch, Günther Ruf

**Affiliations:** 1 Department for General and Visceral Surgery, University Hospital Freiburg im Breisgau, Hugstetter Straße 55, 79106, Freiburg im Breisgau, Baden-Württemberg, Germany; 2 Department for General and Visceral Surgery and Coloproctology, County Hospital Emmendingen, Emmendingen, Germany; 3 Department for General and Visceral Surgery and Coloproctology, Hospital Rosenhöhe Bielefeld, Bielefeld, Germany

**Keywords:** Fecal incontinence, Prosthetic sphincter, Treatment option

## Abstract

**Background:**

Fecal incontinence is a common and severely disabling disorder. For patients with severe fecal incontinence, surgery may prove to be the only adequate treatment option.

**Methods:**

This study reports on 43 patients that were treated with a prosthetic sphincter system between 2005 and 2009 in three coloproctological centres. Main Outcome Measures: complications, anal pressures before and after surgery, fecal continence score.

**Results:**

The new artificial sphincter system significantly improves continence but leads to some complications in clinical practice. After implantation of the device, continence improved significantly (Keller & Jostarndt continence score 2.6 to 14.3 (P < 0.01)). With the band activated, resting pressure improved significantly as compared to baseline (10.7 mmHg vs. 66.1 mm Hg, P < 0.01). The same holds for anal sphincter squeeze pressure (32.2 mmHg versus 85.9 mm Hg, P < 0.01). Complications occurred in 21 patients (48.8%): 10 surgical and 13 technical. Two patients were affected by both technical and surgical problems. The median time of the occurrence was 3 months postop. In five patients difficulties arose within the first postoperative month leading to explantation of the device in three patients. 90% of complications occurred in the first year.

**Conclusions:**

The soft anal band of AMI (AAS), a new artificial anal sphincter, improves severe anal incontinence, but it must be regarded as a last treatment option to avoid a stoma.

## Background

In adult females, the most frequent cause of fecal incontinence is obstetric trauma [1].

Fecal incontinence is usually an acquired disorder. A small percentage of cases are congenital disorders, such as imperforate anus, rectal agenesis, cloacal defects, myelomeningoceles, and meningoceles [2].

The prevalence of fecal incontinence is difficult to determine because patients are reticent to report the disorder. Previous studies estimate the prevalence to be between 2.2 and 20.7 percent [3-6].

Conservative measures, such as dietary and lifestyle modifications and pelvic floor exercises are effective in managing mild symptoms and can also be implemented following surgical procedures [2,7,8]. However, surgery is often the only effective treatment for severe incontinence.

In refractory disease, an artificial sphincter should only be considered as a last resort as an alternative to a stoma. Anal sphincter prostheses are strictly mechanical devices and were first implanted to manage urinary incontinence in 1973 [9]. Fourteen years later, the Lancet reported on a modified “scott-sphincter” to treat fecal incontinence [10]. The main purpose of artificial sphincters is to mimic the external anal sphincter’s (EAS) ability to control the passage of faeces through the anus. They do so with the help of circular, flexible and refillable cuffs. Currently, two systems are being implanted: the Acticon Neosphincter® (Artificial Bowel Sphincter (ABS)) produced by American Medical Instruments (A.M.S., USA) and the Soft Anal Band System produced by Agency for Medical Innovations (A.M.I., Austria).

**Figure 1:**
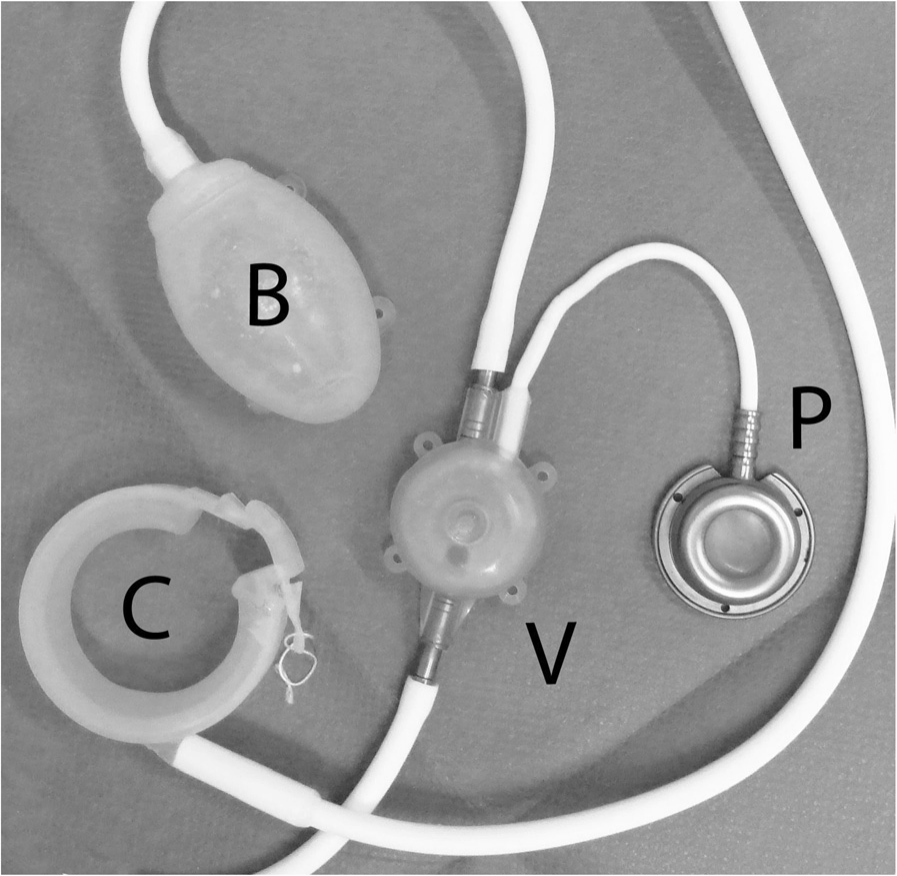
**Components of the soft anal band system ®. P**: calibration port, **V**: valve, **B**: balloon / activator, **C**: cuff ring.

The Artificial Anal Sphincter (AAS) from A.M.I. is similarly designed to the one of AMS (ABS). The AAS is implanted subcutaneously and consists of four parts, which are all connected by silicone tubes (Figure 1). When pressure is applied to the balloon by the patient, the fill-liquid moves from the balloon to the band and continence is achieved. To open the sphincter for defecation, the patient presses on the valve, the fill-liquid moves from the band to the balloon leaving the sphincter open. The connection between the valve and the subcutaneous safe puncture port (semi-automatic pump), which is placed lateral to the iliac crest, permits accurate adjustment of the amount of liquid inside the system (Figure 1). The aim of the study was to determine the effects of the implant on the fecal incontinence and surgical complication rates.

## Methods

At three different coloproctological centres, 43 patients with Grade III fecal incontinence (FI) received a Soft Anal Band System (AAS; Agency for Medical Innovation (AMI), Feldkirch (Austria), CE Body Number (0297) ID: 170530317; Reg. No. 066924 MR2).

Our patient sample included 43 patients, 16 men (average age 54 years; SD 17.6) and 27 women (average age 62 years, SD 9.7). The causes of FI were hereditary (anal atresia) or acquired sphincter defects (mostly due to a trauma), and the sequelae of rectal surgery (see: Table 1). Before surgery all patients were affected by Grade III fecal incontinence (FI) (mean KJCS 2.6 (SD2.3)). Mean follow-up time was 32.3 months (range 1 – 58; SD16.9).

**Table 1:** Causes of fecal incontinence

**Fecal incontinence**			
	**n**	**Female**	**Male**
		**n**	**n**
	43	27	16
inborne / acquired sphincter defect	17	11	6
birth trauma (6x), state after previous surgery (9×): sphincter or pelvic floor repair, rectopexy, hemorrhoidectomy, etc.			
sequelae after rectal surgery:	8	3	5
rectum resection (7×), abdomino-perineal pull-through			
idiopathic	6	6	
(unknown cause)			
neuogenetic	6	5	1
neuroborreliosis, herniated disc, spina bifida, incomplete spinal cord injury, encephalitis disseminata, lesion of pudendal nerve			
pelvic / pelvic floor trauma (road accident)	3		3
Other	3	2	1
scleroderma (2×), irradiation of prostate cancer			

Preconditions for implantation of an artificial sphincter were that all other therapeutic options had failed, that the soft tissues around the anus were intact and patient compliance was adequate. Contraindications were chronic inflammatory bowel syndromes, therapy-resistant diarrhoea, irrigation therapy, receptive anal intercourse, pregnancy, inverse acne, tight (fibrotic) anal canal and psychiatric disorders. Anal or rectal prolapse was treated prior to artificial sphincter implantation. The data were collected prospectively at each centre. The following outcome variables were recorded: complications: major surgical complications such as infection, bleeding, penetration and technical problems such as impaired function of the implant, handling problems. Patient surveillance was carried out locally at the respective centres based on local surveillance standards. The prosthesis was viewed as the last treatment option before the formation of a definitive stoma. All patients gave informed consent. Continence was measured with a standardized questionnaire (Keller & Jostarndt continence score (KJCS)) [11]. The score consists of 10 items, which are scaled from 0 to 6 points. The total score has a range of 36–0 points. The scale is divided into ranges to indicate four different degrees of continence (see Table 2). A score between 11 and 0 corresponds to Grade III incontinence.

**Table 2:** Keller & Jostarndt continence score (KJCS)

**Symptom**	**0 point**	**1 point**	**2 points**
frequency	> 3 per day	2-3 per day	0 - 1 per day
consistency	predominant liquid	predominant mushy	perdominant solid
perception of urgency	regularly none or too late	uncertain	always just in time or certain
lead time	none	seconds	minutes
discrimination (gaseous, liquid, solid)	none	uncertain	certain
care needs	always	occasional	none
**Symptom**	**0 point**	**3 points**	**6 points**
soiling	always	occasional	none
incontinence: gas	always	occasional	none
incontinence: liquid	always	occasional	none
incontinence: solid	always	occasional	none
total score			
36 – 31 points	30 – 24	23-12	11 - 0
grade 0	grade I	grade II	grade III
complete continent	little contamination	gross contamination	complete incontinence

The KJC scores were calculated before the operation and at the time of activation of the device. Anal resting and squeeze pressures (RP / SP) were measured using a water-filled pressure sensor that was connected to a handheld device designed to display and record values (Sphinctometer: STM-0169-SM, smProMedico, Aachen, Germany).

Measurements were recorded before surgery and at the time of system activation. Readings were taken for the filled (active) and relaxed (inactive) anal band.

Statistical analysis was performed with STATA® 11.0 statistical packages.

### Soft anal band system

Currently, two systems are being implanted: the Acticon Neosphincter® (Artificial Bowel Sphincter (ABS)) produced by American Medical Instruments (A.M.S., USA) and the Soft Anal Band System produced by Agency for Medical Innovations (A.M.I., Austria).

The Artificial Anal Sphincter (AAS) from A.M.I. is similarly designed to the one of AMS (ABS). The AAS is implanted subcutaneously and consists of four parts, which are all connected by silicone tubes: 1.) the Soft Anal Band made of silicone (available in three sizes, plus two extension parts, also available in two different sizes), 2.) a silicone-bonded valve to control the fill-liquid of the system, 3.) a manually operated silicone balloon (activator) to move the fill-liquid into the band to close the sphincter, and 4.) a titanium port with anti-kink protection for individual adjustment of the fill volume. When pressure is applied to the balloon by the patient, the fill-liquid moves from the balloon to the band and continence is achieved. To open the sphincter for defecation, the patient presses on the valve, the fill-liquid moves from the band to the balloon leaving the sphincter open. The connection between the valve and the subcutaneous safe puncture port (semi-automatic pump), which is placed lateral to the iliac crest, permits accurate adjustment of the amount of liquid inside the system (Figure 1).

### Surgery

**Figure 2:**
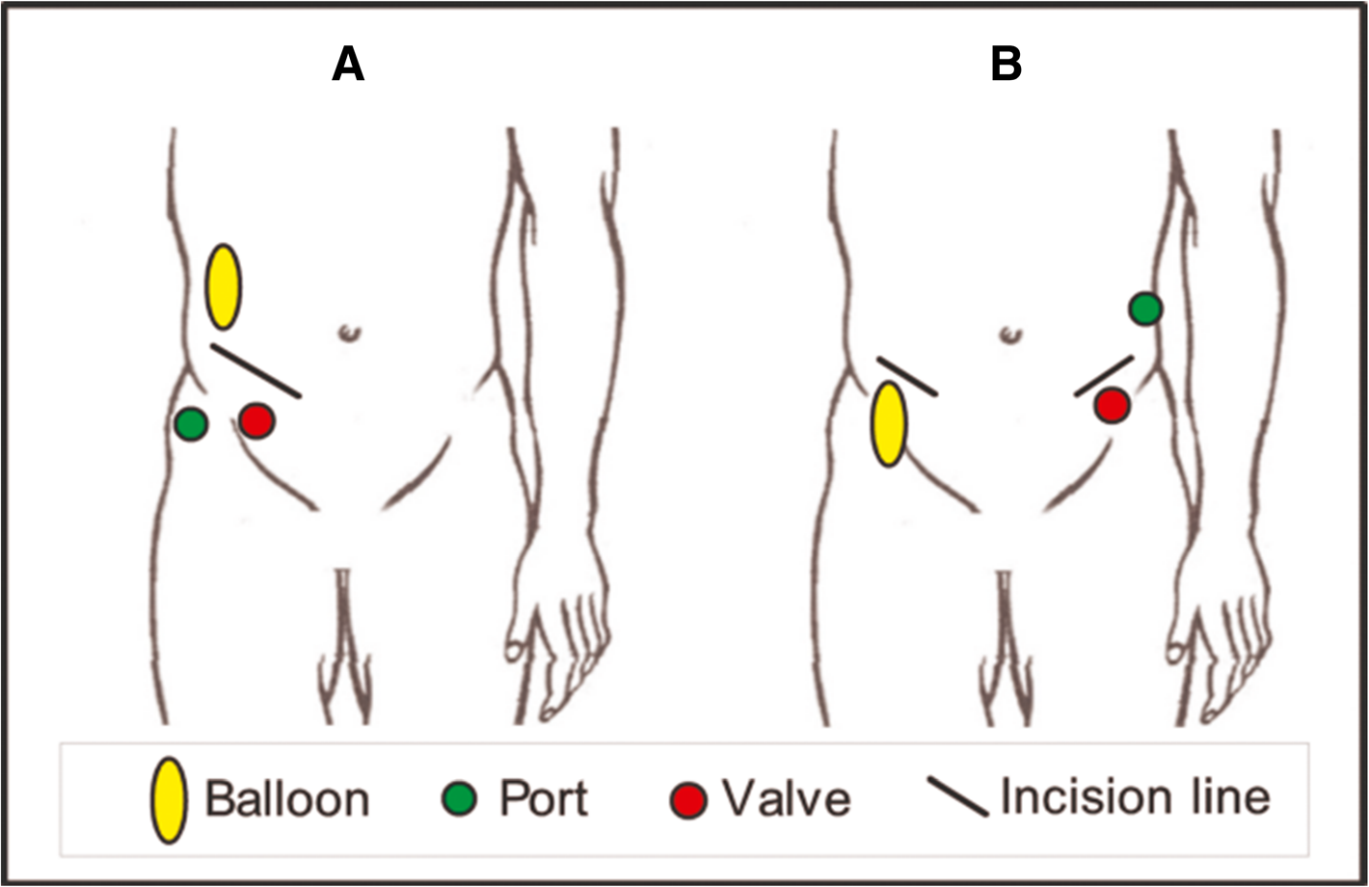
**Two possible implantation sites for the system’s components.** The implant positions must be discussed with the patient and accessability and operation of the elements has to be simulated. **A**: The balloon (activator), port and valve are placed on the right side of the abdomen through a single portal. The valve is placed right above the iliac crest, the activator (balloon) over the fascia of the abdominal muscles in the subcutaneous tissue. The port is positioned for easy access through a puncture in the subcutaneous tissue. **B**: Two incisions in the right and left lower abdomen: Activator (balloon) and valve are placed above the contralateral iliac crest. The port is placed close to the valve in the subcutaneous tissue.

All patients received bowel preparation and prophylactic antibiotics before surgery. Patients were placed in the lithotomy position, and skin incision lines were marked by the surgeon according to anatomical landmarks and according to the chosen implantation position (Figure 2). After bilateral incision 3–4 cm from the anocutaneous line, a circumferential, subcutaneous pocket is created by dissection of the ischiorectal space.

The size of the anal band was either estimated or measured with a tape measure. After positioning the anal band, the connecting tube to the valve was placed subcutaneously from the perianal region to the lower abdominal wall. The perianal wounds were closed with sutures in double layer technique or sealed with histoacrylic glue.

The tube from the band connects on one side of the valve. The activator and the port are also connected to the valve. Once in situ, the system was deflated and then filled with a defined mixture of 18 ml distilled water and radio-opaque solution. The system was now checked for function and leakage. Anal band function was tested manometrically and digitally and then left in the open position for 5–6 weeks to allow for healing.

Valve, activator and port were fixed with PDS 2/0 to the underlying fascia. After five to six weeks, patients were instructed on how to activate the Soft Anal Band System.

This observational study was approved by the ethics committee of the university hospital of Freiburg (No. 437/129).

## Results

Anal pressure values changed significantly after the operation. On average 10.7 mmHg (SD8.3) was measured for resting pressure, and 32.2 mmHg (SD21.6) for squeeze pressure.

After surgery, continence improved significantly (KJCS 2.6 (SD2.3) to 14.3 (SD2.7), P < 0.01).

With the band activated, resting pressure improved significantly as compared to baseline (10.7 (SD8.3) vs. 66.1 (SD14.3), P < 0.01). The same holds for squeeze pressure (32.2 (SD21.6) vs 85.9 (SD21.4), P < 0.01). Inactivation of the band led to a significant drop in pressure values: 66.1 (SD14.3) vs. 16.9 (SD6.2) (P < 0.01) for resting pressure, 85.9 (SD21.4) vs 40.7 (SD17.0) (P < 0.01) for squeeze pressure (see Table 3).

**Table 3:** Anal resting and squeezing pressures before and after surgery

	**Baseline**	**Pressures at the time of activation**		**P**
		**Filled cuff**	**Relaxed cuff**	
**resting pressure (SD) [mmHg]**	10.70 (8.32)	66.10 (14.27)	16.93 (6.15)	(*)
**squeezing pressure (SD) [mmHg]**	32.21 (21.58)	85.91 (21.44)	40.65 (17.03)	(*)

(*) All differences between the mean values were highly significant at P < 0.01.

(*) Baseline: value before the operation.

(*) Time of activation = 5–6 week after the operation.

### Surgical outcome

Complications occurred in 21 cases (48.8%): 10 surgical and 13 technical problems (failure of prosthetic components or failure related to handling) occurred. Two patients were affected by both technical and surgical problems. The median period for complications was 3 months. 5 times there were early problems within the first month: leading in 3 cases (infection 2×; perforation of device 1×) to the removal of the devices. 90% of the problems occurred in the first year. In 9 patients (21%) the system had to be explanted due to infection (4×), perforation (1×), tumor recurrence (1×), rectovesical fistula (1×), ineffective treatment (1×) or pain (1×). In 3 of theses patients (1× perforation, 2× infection) the system was re-implanted successfully and without further complications.

10 Revisions of the device were necessary due to failure of the valve (8×) and failure of the anal band (2 ×). In all cases the affected components were replaced. Two patients had to be treated repeatedly due to valve failures.

In 4 patients revisions where necessary due to development of a fibrotic capsule (1 ×); weight gain (1× - > transposition of the device); pain (1×); bleeding after activation of the device occurred once under oral anticoagulation. In all cases the affected implant site was surgically revised, the implants were retained.

Two patients were unable to operate the valve by themselves. Close relatives had to help in handling the valves.

## Discussion

In discussing the results, one must bear in mind that prosthetic sphincters are the last therapeutic option in the treatment of severe fecal incontinence before a stoma is created. A stoma is for many patients a source of significant psychological distress. In order to avoid a stoma, these patients are willing to live with an artificial implant, which may only improve their symptoms slightly. In the study present 43 patients were treated with a new artificial bowel sphincter and prospectively examined over 32 month on average. The AAS elevates anal pressure values significantly from 11 to 66 mmHg for the resting pressure and 32 to 85 mmHg for the squeezing pressure respectively. Although many patients are still significantly incontinent, the condition is less severe. KJC-Scores improved from 3 to 14, which corresponds to an FI grade II.

The modest prospects of treatment must be given to the patient.

**Figure 3:**
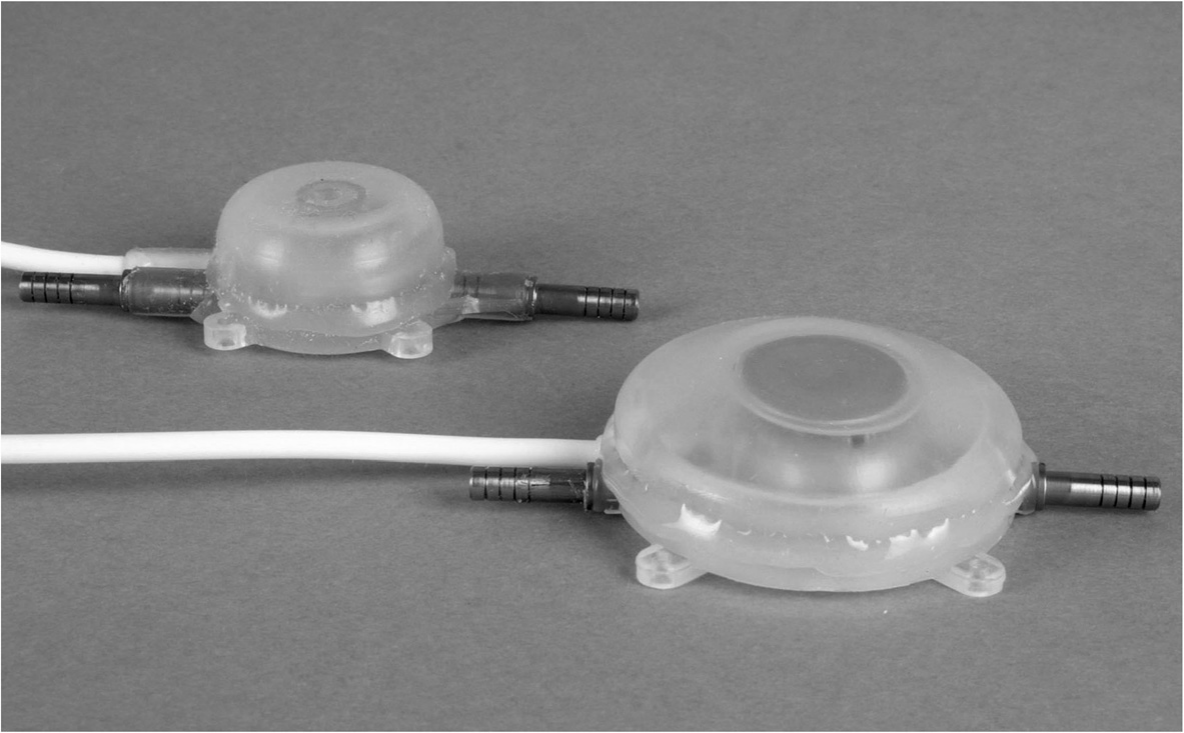
Valve after revision (right hand side) compared to the old one (left side).

In terms of technical function and complications, the AMI Soft Anal Band system (AAS) should be compared with the current published results for the best investigated artificial sphincter system for fecal incontinence, namely, ABS Neosphincter®. With respect to wound infection and penetration rates our study provided some initial indications: Wound infection rate was 9% (4 out of 43 patients), which is in the lower range compared to the reported rates for the Acticon Neosphincter® [12-23]. In the population of our study, only 1 of 43 treated patients experienced a penetration of the Soft Anal cuff ring into the rectum. For the Neosphincter penetration rates up to 25% are described [18,23]. Overall the prosthesis had to be removed in 9 cases (21%) for various reasons and was successfully re-implanted in three cases. In eight cases revisions were necessary to deal with a dysfunctional valve. These complications occurred mainly during the initial implantation procedures between 2005 and 2006. Experience with the system and technical modifications of the valve by the manufacturer reduced those particular problems. Nevertheless, handling the manually operated parts of the system, especially the valve, remained a problem for the patients and two patients needed constant support from their relatives. The systems are designed to be operated through the patient’s abdominal wall, which may be thick in the obese patient. A balloon the size of a tangerine has to be pressed together through the fatty tissue of the abdominal wall and this action may be painful and may cause the activator to shift, slip or distort. The valve cannot be positioned on a bony support for functional reason, therefore, the fascia and the tensed abdominal wall are the only mechanisms of counter pressure. The valve’s pressure point is less than 1 cm^2^, which is very small and not easily located. This small pressure point must be accurately depressed through a layer of cutis and subcutaneous tissue and a large force applied to open the valve, which requires good hand-valve coordination and considerable aptitude on the part of the patient. Reduction of the subcutaneous tissue above the valve can bring relief. The manufacturer is aware of this biomechanical problem and a valve revision was released in 2011 (Figure 3).

### Limitations

The study is of descriptive nature. The results reflect the experiences with this new artificial bowel sphincter device. The data were collected uniformly and prospectively. Although the results are compared with published data for the well-studied Neosphincter, general statements can not be made about an advantage or inferiority of the AAS.

## Conclusion

Innovative sphincter prosthesis has now become available in the form of the soft anal band system (AAS). Infections and penetration rates are comparatively low. Handling the valve is the main product-related difficulty, whereby the product manual does provide adequate instructions. The AAS improves incontinence, but it must be regarded as a last surgical treatment option to avoid a stoma. Preliminary results show a considerable complication rate with wound infections in four and band perforation / penetration in one patient. Explantation of the device was necessary in a total of 9 out of 43 patients.

Technical problems relating to valve handling or system failure should be addressed in the design of the improved second generation Soft Anal Band System (AAS). The manufacturer released a new version of the valve in 2011.

## Abbreviations

KJCS: Keller & Jostarndt continence score; AAS: Artificial Anal Sphincter (Soft Anal Band System ®, A.M.I); ABS: Artificial bowel sphincter (Acticon Neosphincter ®, A.M.S); A.M.I: Agency for medical innovations (Austria); A.M.S: American medical instruments (USA); EAS: External anal sphincter; RP: Resting pressure; SP: Squeezing pressure.

## Competing interests

The authors declare that they have no competing interests.

## Authors’ contributions

MG, BU, LM and RG performed the implantations and collected the data. All five authors drafted and revised the manuscript and gave final approval for the version to be published.

## Pre-publication history

The pre-publication history for this paper can be accessed here:

http://www.biomedcentral.com/1471-2482/13/45/prepub
